# Synthesis and characterization of TiO_2_ nanoparticles combined with geraniol and their synergistic antibacterial activity

**DOI:** 10.1186/s12866-023-02955-1

**Published:** 2023-08-02

**Authors:** Almotasem Bellah Younis, Vedran Milosavljevic, Tatiana Fialova, Kristyna Smerkova, Hana Michalkova, Pavel Svec, Peter Antal, Pavel Kopel, Vojtech Adam, Ludek Zurek, Kristyna Dolezelikova

**Affiliations:** 1 Department of Chemistry and Biochemistry, Mendel University in Brno, Brno, Czech Republic; 2 Department of Inorganic Chemistry, Faculty of Science, Palacky University, Olomouc, Czech Republic

**Keywords:** Titanium dioxide, Nanoparticles, Geraniol, Minimum inhibitory concentration, Biofilm, Methicillin-resistant *Staphylococcus aureus*, MRSA, *Escherichia coli*, SEM-EDS

## Abstract

**Background:**

The emergence of antibiotic resistance in pathogenic bacteria has become a global threat, encouraging the adoption of efficient and effective alternatives to conventional antibiotics and promoting their use as replacements. Titanium dioxide nanoparticles (TiO_2_ NPs) have been reported to exhibit antibacterial properties. In this study, we synthesized and characterized TiO_2_ NPs in anatase and rutile forms with surface modification by geraniol (GER).

**Results:**

The crystallinity and morphology of modified TiO_2_ NPs were analyzed by UV/Vis spectrophotometry, X-ray powder diffraction (XRD), and scanning electron microscopy (SEM) with elemental mapping (EDS). The antimicrobial activity of TiO_2_ NPs with geraniol was assessed against *Staphylococcus aureus*, methicillin-resistant *Staphylococcus aureus* (MRSA), and *Escherichia coli*. The minimum inhibitory concentration (MIC) values of modified NPs ranged from 0.25 to 1.0 mg/ml against all bacterial strains, and the live dead assay and fractional inhibitory concentration (FIC) supported the antibacterial properties of TiO_2_ NPs with GER. Moreover, TiO_2_ NPs with GER also showed a significant decrease in the biofilm thickness of MRSA.

**Conclusions:**

Our results suggest that TiO_2_ NPs with GER offer a promising alternative to antibiotics, particularly for controlling antibiotic-resistant strains. The surface modification of TiO_2_ NPs by geraniol resulted in enhanced antibacterial properties against multiple bacterial strains, including antibiotic-resistant MRSA. The potential applications of modified TiO_2_ NPs in the biomedical and environmental fields warrant further investigation.

**Supplementary Information:**

The online version contains supplementary material available at 10.1186/s12866-023-02955-1.

## Background

Spread of antibiotic-resistant bacteria is a major concern worldwide due to infections leading to high morbidity and mortality rates [1, 2]. The impact of antibiotic resistance on health and disease has been further underlined by recent developments in understanding the microbiome [3–5].To combat this issue, alternative compounds such as natural products and nanoparticles have been explored [6]. Metallic nanoparticles (NPs) such as zinc, cerium, aluminum, nickel, zirconium, magnesium, and titanium dioxide have the potential to act as antimicrobial agents due to their small size and high surface-to-volume ratio [7–11]. In particular, titanium dioxide has been shown to have enhanced photoelectrochemical performance and effective photocatalytic properties [12, 13] These properties allow them to generate reactive oxygen species (ROS), such as hydrogen peroxide, hydroxyl radicals, and superoxide anions, that can penetrate bacterial cell walls and disrupt cell functions, leading to cell death [14–16]. Additionally, NPs can be modified with organic compounds to enhance their antimicrobial activity, making them promising candidates for treating infections caused by antibiotic-resistant bacterial strains [17, 18]. One of the NPs that has gained attention for its antimicrobial properties is titanium dioxide (TiO_2_) [14].

Titanium dioxide (TiO_2_) is a unique material that displays various physical and chemical characteristics, including the ability to produce ROS when exposed to light, creating pairs of holes. These electron-hole pairs can react with water or oxygen and, as a result, generate ROS [19, 20] Multiple studies have demonstrated that TiO_2_ exhibits good antibacterial and antifungal activity against a broad range of Gram-positive and Gram-negative bacteria [21]. TiO_2_ has three different crystalline phases: rutile, anatase, and brookite; each can exhibit metastable behavior as various factors affect its crystal shape and cause a possible conversion among the three phases [22, 23]. It was shown that anatase and rutile phases are stable and have great photocatalytic activity [24–26]. The cytotoxicity of TiO_2_ varies depending on the crystal shape, as results of several studies showed that the toxicity of TiO_2_ was dependent on the concentration and the crystal phase; therefore, the response of bacteria to the crystals can be different [16, 27–29].

Using natural compounds can greatly enhance the inherent physical and chemical properties of NPs [30, 31]. For instance, TiO_2_ has been used as an antimicrobial additive in polyamide 12 powder, and nitrogen-doped titanium dioxide nanoparticles have demonstrated photoinduced antimicrobial properties under visible-light irradiation [32, 33] The small size, high surface energy, and strong hydrophilicity for surface hydroxylation may increase TiO_2_ NPs compatibility with polymer materials [34, 35]. Incorporating inorganic NPs into organic polymeric nanocomposites is a promising strategy to enhance their antimicrobial properties [36–38]. For example, monoterpenes incorporated with TiO_2_ had a greater antimicrobial effect [39]. Monoterpenes are natural products containing active compounds such as phenols, flavonoids, and terpenoids. Geraniol is a mono-terpenoid with low toxicity and high solubility, and when combined with TiO_2_ NPs, it may exhibit enhanced antimicrobial properties [40, 41].

The objectives of this study were to: (a) synthesize TiO_2_ NPs and combine them with geraniol; (b) assess the antimicrobial activity of this material against planktonic and biofilm-forming bacterial cells. This is a novel study focusing on the combined effect of geraniol and TiO_2_ NPs and their synergistic activity against *E. coli, Staphylococcus aureus*, and MRSA. This distinguishes it from the previous studies that have mainly focused on the effects of individual agents.

## Results

### Characterization of the TiO_2_ NPs

**Fig. 1:**
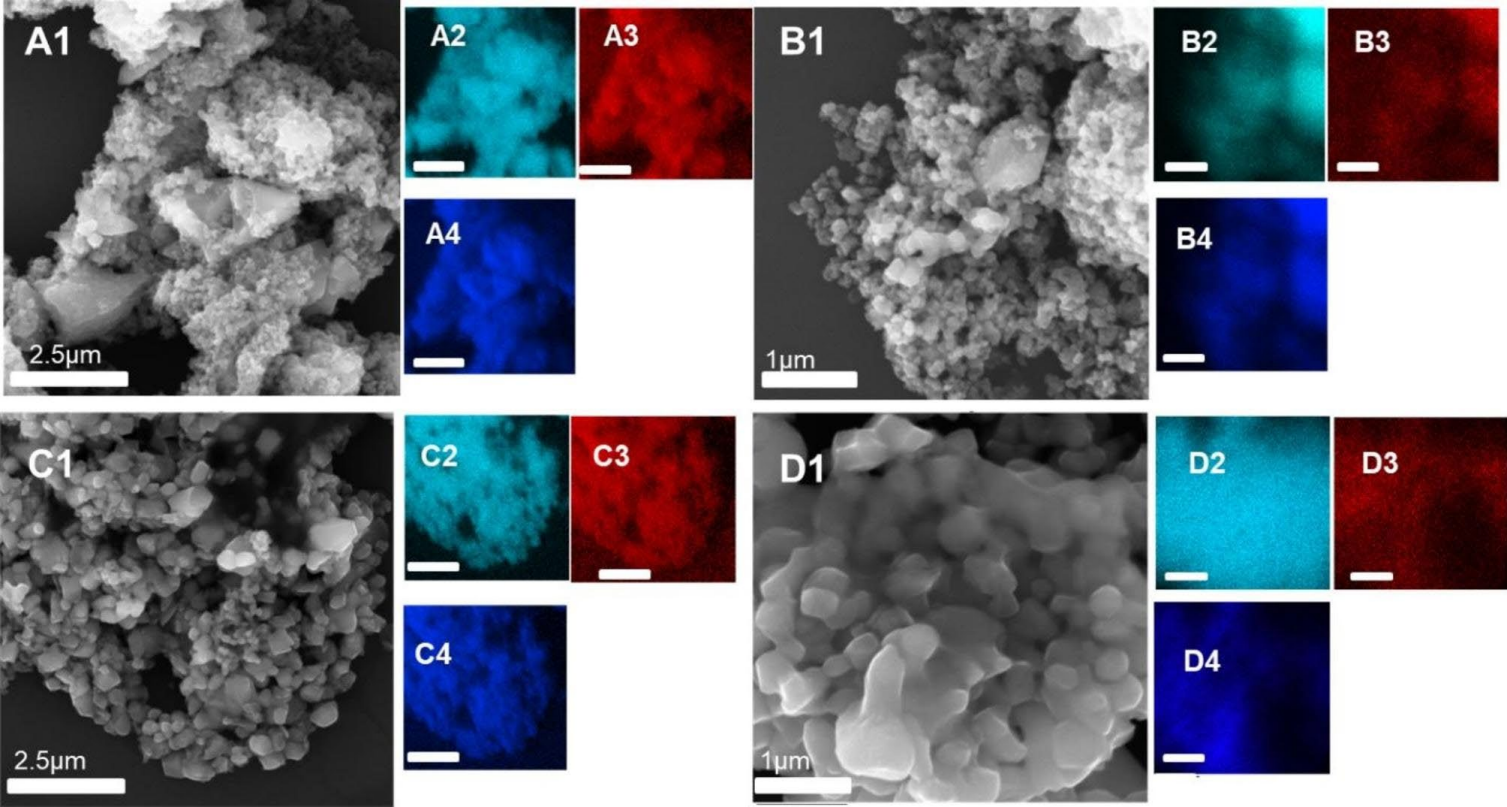
SEM images of unmodified and modified TiO_2_ NPs. A1-A4 unmodified anatase TiO_2_ NPs and their corresponding surface elements (Ti, C, and O, respectively). B1-B4 unmodified rutile TiO_2_ NPs and their corresponding surface elements (Ti, C, and O, respectively). C1-C4 and D1-D4. Modified anatase and rutile TiO_2_ NPs and their corresponding surface elements (Ti, C, and O, respectively).

Synthesized TiO_2_ NPs and their combination with GER were characterized through elemental analysis, and GER was detected on the surface of TiO_2_. Scanning electron microscopy revealed the size, shape, and surface morphology of the anatase and rutile TiO_2_ material and showed that NPs were successfully formed. The anatase form of the TiO_2_ had clusters of particles with an average size of 300 ± 100 nm. High-magnification imaging revealed dense nanocrystalline domains on the surface. The anatase form of the TiO_2_ with GER resembled unmodified TiO_2_ NPs, did not vary in size after surface modification with GER, and showed a strong tendency for aggregate. However, imaging revealed nanocrystalline domains and a relatively smooth surface (Fig. 1A, B), compared to the unmodified form of TiO_2_ indicating the presence of GER on the surface. In the rutile form of TiO_2_ without surface modification (Fig. 1A, B), the nanoparticles (NPs) had irregular rock shapes with an average size of 100 ± 10 nm and a tendency to aggregate. The same properties were observed for the rutile form of the TiO_2_ NPs modified with GER. However, in the case of the rutile form, the surface was not smooth as it did in the case of the anatase form after modification with GER. To further confirm the presence of GER on the surface of TiO_2_ NPs, we used the energy-dispersive X-ray spectroscopy (EDS) for an elemental mapping analysis. The elemental dispersion on the TiO_2_ surface in both forms showed the presence of carbon, which confirms the successful modification with GER. Both non-modified forms also showed signs of carbon; however, in trace amounts (contamination for ambiance) only (Fig. 1).

**Fig. 2:**
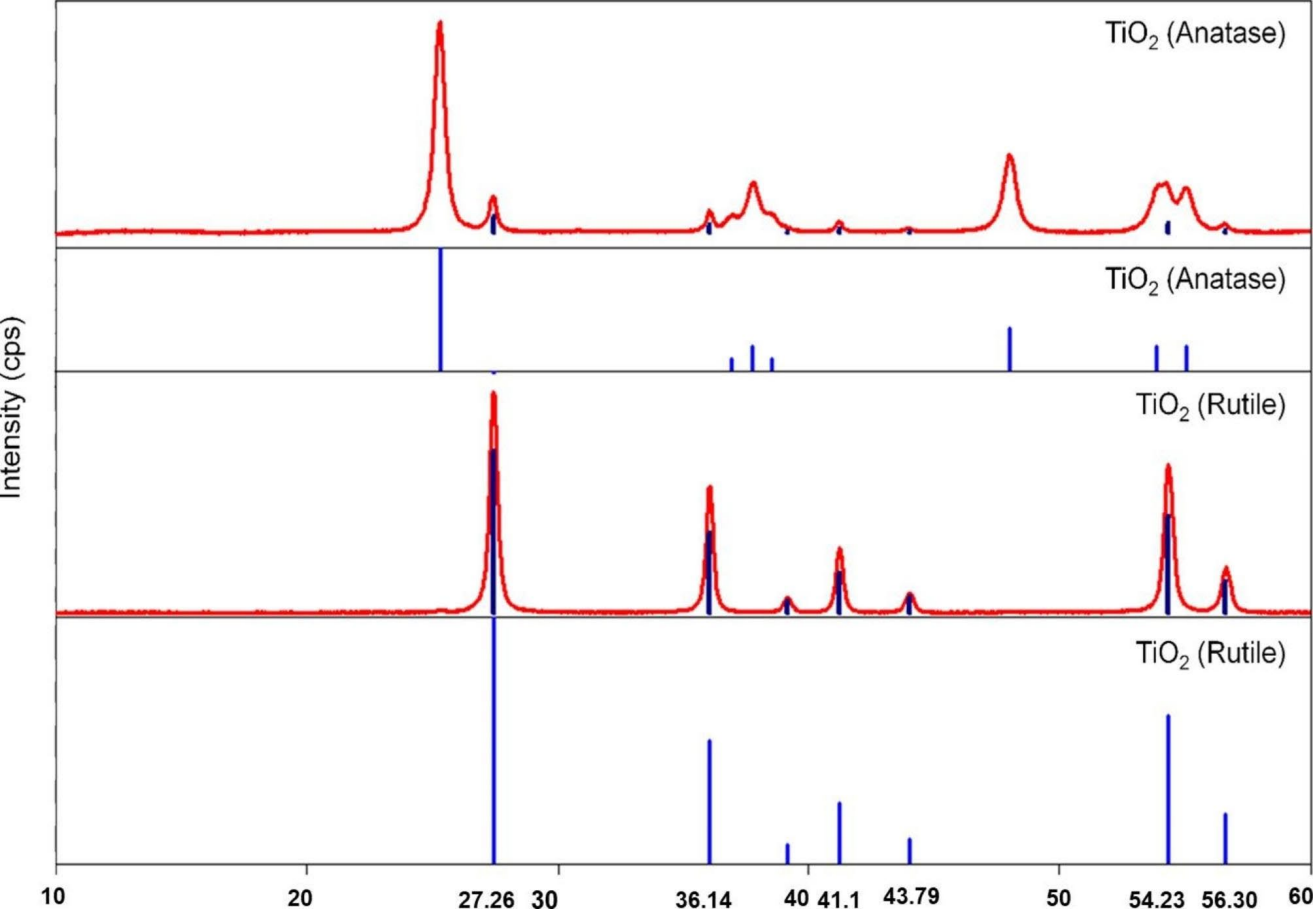
X-ray powder diffraction patterns of anatase and rutile form of TiO_2_.

X-ray crystallography (XRD) patterns confirmed the crystallinity of the TiO_2_ (anatase and rutile) observed under SEM. These patterns showed that both forms of TiO_2_ NPs were polycrystalline with well-defined peaks indicating a high degree of crystallinity. The XRD pattern exhibited diffraction peaks at 27.26°, 36.14°, 41.16°, 43.79°, 54.23°, 56.30°, 62.92°, and 68.67°, which belongs to the anatase and tetragonal rutile form of TiO_2_ NPs [42, 43] (Fig. 2). These results indicate that increasing the temperature without using any additive changed the biphasic anatase/rutile to rutile TiO_2_ NPs.

**Fig. 3:**
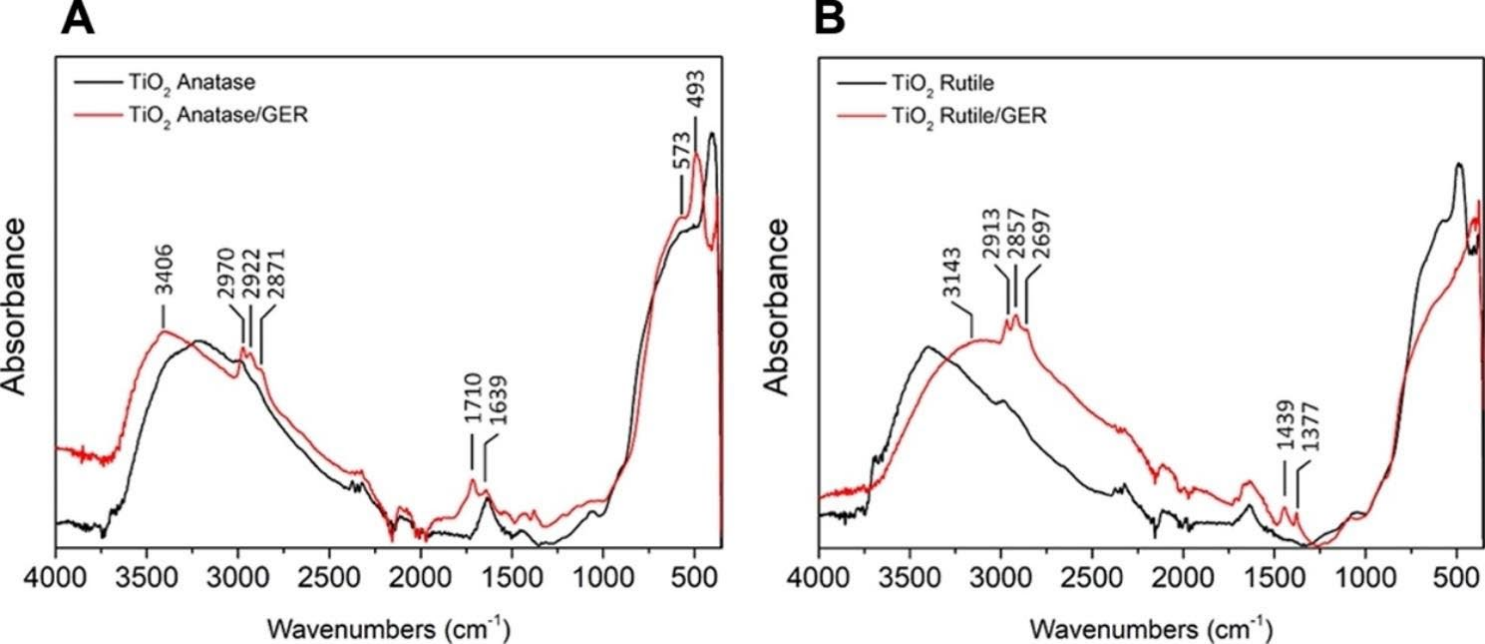
Baseline-corrected FTIR spectra of the TiO_2_/anatase and TiO_2_/anatase/geraniol (**A**) and TiO_2_/rutile and TiO_2_/rutile/geraniol (**B**) in the 4000 to 400 cm^–1^ range

To confirm the presence of GER on the surface of TiO_2_ NPs, we also performed FTIR analysis (Fig. 3). The data revealed the presence of vibrational peaks that belong to organic functional groups, indicating the presence of GER. In particular, the broadband in the range of 3.500–3.000 cm ^− 1^ was observed in the anatase and rutile TiO_2_ NPs, which is related to the stretching of hydroxyl groups (O–H), indicating the presence of surface water as moisture (Fig. 3A and B). The FTIR spectrum of anatase TiO_2_ NPs (Fig. 3A) showed a typical band on 1630 cm ^− 1^ that is attributed to the H–O–H vibrations from physisorbed water [19, 20] and an absorption band detected at 438 cm ^− 1^ that belongs to the Ti–O–Ti vibrations related to the anatase phase [21, 22]. Similar absorption band peaks were observed in the case of rutile TiO_2_ NPs (Fig. 3B), with the exception of the Ti–O–Ti vibration, which is shifted and detected at 512 cm ^− 1^ as a result of the temperature increase (900 °C) during rutile preparation [18, 21]. Investigating the molecular vibration features of the GER-modified TiO_2_ NPs, we detected additional peaks indicating successful surface modification. In the case of anatase TiO_2_ NPs (Fig. 3A), the vibrational peaks detected at 3406 cm ^− 1^ and 1639 cm ^− 1^ are related to the O–H groups coming from surface and physisorbed water, similar to the case of unmodified TiO_2_ NPs. The peaks detected at 2970 cm^− 1^, 2922 cm^− 1^, and 2871 cm^− 1^ belong to the CH stretching vibrations of GER [19, 23], while the stretching vibrations at 1710 cm^− 1^, corresponding to the carbonyl group (C = O), confirm the consumption of hydroxyl groups from GER during surface modification of TiO_2_ [24, 25]. The stretching of other peaks at 493 cm^− 1^ and 573 cm^− 1^ belongs to TiO_2_ [18]. A similar position of detected peaks can be observed in the case of rutile TiO_2_ NPs modified with GER (Fig. 3B). The only exception is the deformed C–H bending vibrations detected in two peaks at 1377 cm^− 1^ and 1439 cm^− 1^, which belong to the methylene (–CH_2_–) and methyl (–CH_3_) groups [24], confirming the interaction of GER with TiO_2_ NPs.

In addition to the analyses described above, the FTIR spectra of free GER and GER-modified TiO_2_ NPs were examined, and the results showed notable alterations in peak locations indicating GER-TiO_2_ interaction (Figure S1). For instance, in the rutile and anatase GER-modified TiO_2_ NPs, the O-H stretching vibration peak changed from 3.330 cm^− 1^ in free GER to 3.406 cm^− 1^ and 3.143 cm^− 1^, respectively. Similar considerable changes in the C-H bending and stretching vibrations of the -CH_2_ and -CH_3_ groups were seen after surface modification. These peak shifts show the impact of GER-TiO_2_ interactions on the vibrational behavior of these functional groups and confirm the effective surface modification of TiO_2_ NPs with GER. Additional characteristics of our synthesized TiO_2_ nanoparticles, including particle size distribution and zeta potential, are further explored in the supplementary materials (Figure S2 and S3).

### Effect of TiO_2_/GER treatments on bacteria

**Fig. 4:**
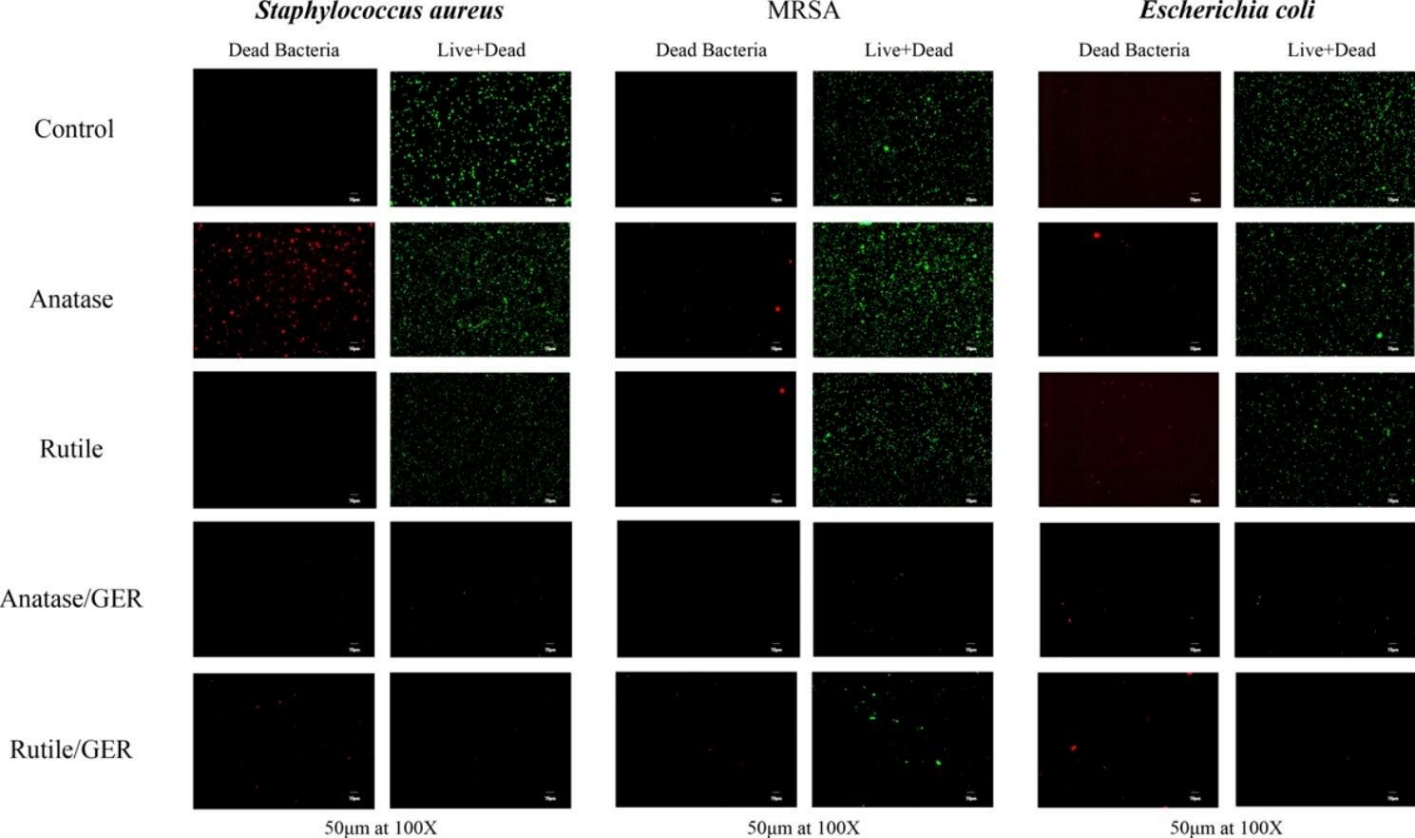
The LIVE/DEAD cell images of *S. aureus*, MRSA, and *E. coli* treated with TiO_2_ anatase, TiO_2_ rutile, TiO_2_ /anatase/GER, and TiO_2_ /rutile/GER—magnification 100×. The scale bar is 50 μm. The saturation was processed equally across all micrographs

It was observed that both TiO_2_/anatase/GER and TiO_2_/rutile/GER exhibit activity against *S. aureus*, MRSA, and *E. coli*, with MIC values ranging from 0.25 to 1.0 mg/ml (Table 1). In contrast, anatase and rutile forms alone did not display any strong bactericidal properties, and GER displayed indifferent activity with MIC values of 4.25 mg/ml The interaction between GER and TiO_2_ inhibitory was confirmed by the FIC index values, which showed additive effects in the case of MRSA with a value of 0.735294 and synergism for *S. aureus* and *E. coli* with values of 0.284314 and 0.22549 respectively (Table 1). Furthermore, the supporting data can be found in (Figure S4, and Tables S1 and S2). Live and dead assays showed that the viability of *S. aureus*, MRSA, and *E. coli* was significantly reduced after treatment with both TiO_2_/anatase/GER and TiO_2_/rutile/GER. At concentrations of 0.25 mg/ml and 0.5 mg/ml, dead cells (stained in red) and live cells (stained in green) were barely visible, indicating that the treatment had a bactericidal effect on all strains, including the MRSA (Fig. 4). Images of control samples showed an increase of live cells (stained in green) and a decrease in dead cells (stained in red).

**Table 1:** Minimum inhibitory concentration (MIC) and FIC index of GER, anatase, and rutile and their combination against various bacterial strains

Bacteria	Component	MIC (mg/ml)	FIC Index	Description
MRSA	Anatase	2	NaN	Indifference
MRSA	Rutile	2	NaN	Indifference
MRSA	GER	4.25	NaN	Indifference
MRSA	Anatase/GER	1	0.735294	Additive
MRSA	Rutile/GER	1	0.735294	Additive
*S. aureus*	Anatase	1.5	NaN	Indifference
*S. aureus*	Rutile	1.5	NaN	Indifference
*S. aureus*	GER	2.125	NaN	Indifference
*S. aureus*	Anatase/GER	0.25	0.284314	Synergism
*S. aureus*	Rutile/GER	0.25	0.284314	Synergism
*E. coli*	Anatase	1.5	NaN	Indifference
*E. coli*	Rutile	1.5	NaN	Indifference
*E. coli*	GER	4.25	NaN	Indifference
*E. coli*	Anatase/GER	0.25	0.22549	Synergism
*E. coli*	Rutile/GER	0.25	0.22549	Synergism

* Note: “NaN” in the FIC index column indicates that the Fractional Inhibitory Concentration (FIC) was not calculated for the respective component against the particular bacterial strain

### Biofilm assay

**Fig. 5:**
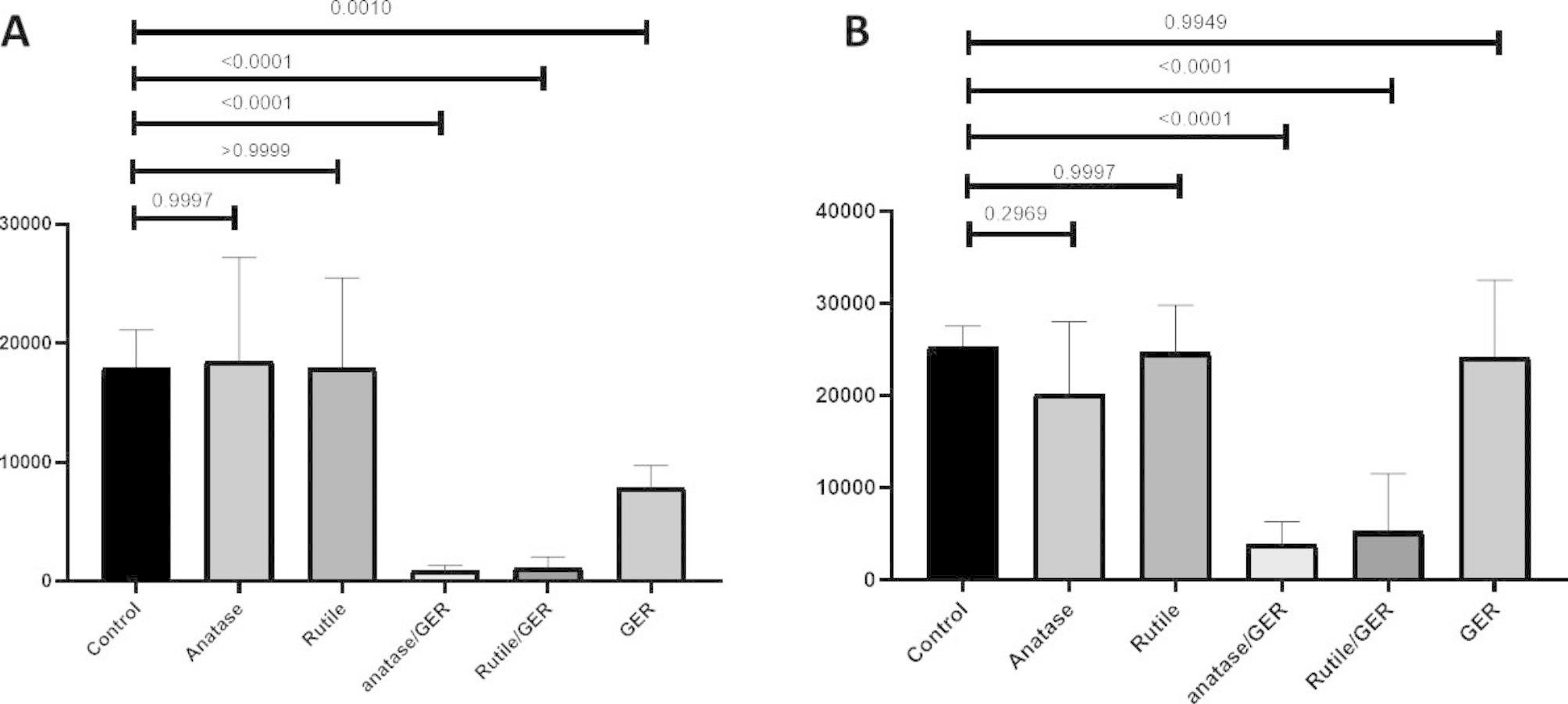
Fluorescence intensity (a.u.) of bacterial biofilm: Efficiency of modified and non-modified TiO_2_ against MRSA biofilm with UV-irradiation **A**) and without UV irradiation **B**).

It was observed that TiO_2_/anatase/GER treatment was highly effective against the MRSA biofilm, reducing its growth by 84.6% (p < 0.0001). Similarly, the TiO_2/_rutile/GER treatment reduced the biofilm growth by 80% (p < 0.0001). We also measured the effect of UV light (c 280 nm) under the same conditions and found that after 60 min, the results were similar to the non-UV light treatment, with the exception of a significant reduction in viability after geraniol treatment compared to that of the negative control (bacteria alone) **(**Fig. 5A and B).

**Fig. 6:**
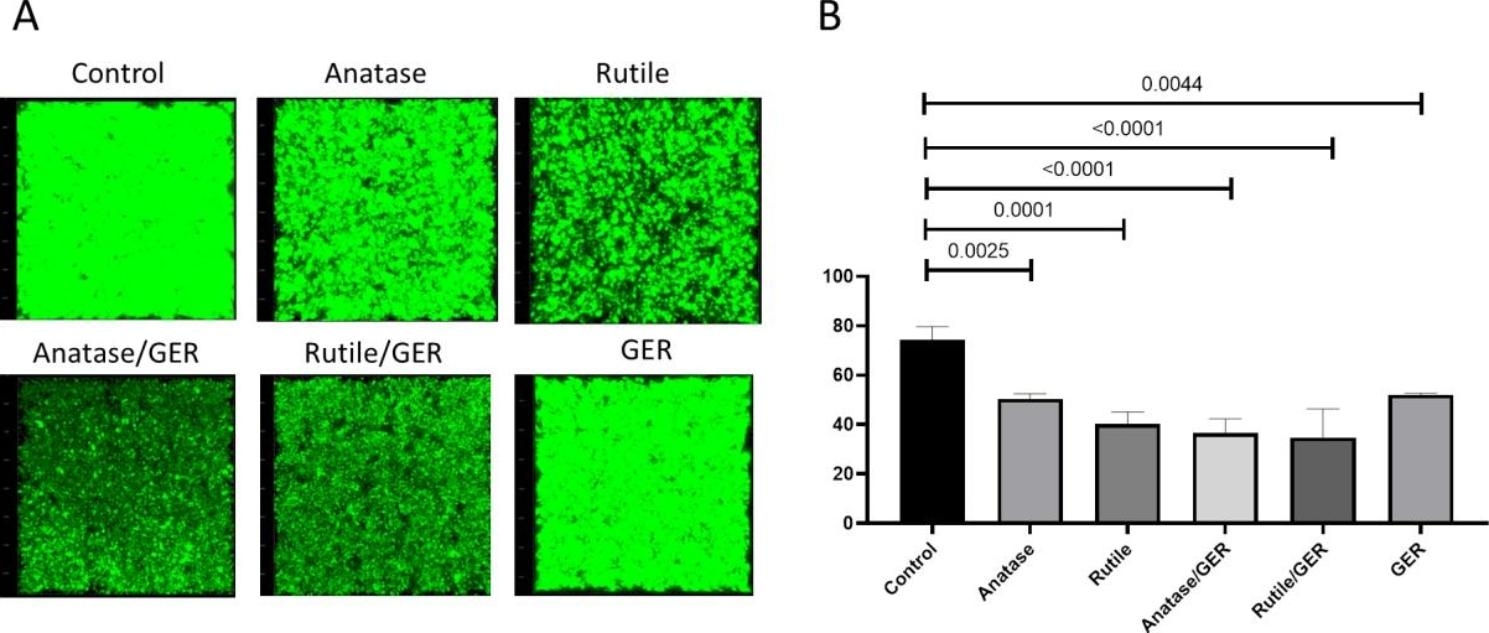
**A)** 3D images from confocal laser scanning microscopy of cells in biofilm with intact membranes (green) and disturbed bacterial biofilm (black areas). **B**) Biofilm thickness after different treatments.

Confocal laser microscopy was employed for a more qualitative, in-depth evaluation of the mixed TiO_2_ nanoparticles (NPs) with GER on MRSA biofilm. By analyzing fluorescence intensity distribution, it was found that both TiO_2_/anatase/GER and TiO_2_/rutile/GER significantly reduced biofilm thickness to values less than 30,000 nm compared to that of the control (p < 0.0001) (Fig. 6A). Interestingly, the treatment with anatase and rutile forms alone also decreased the biofilm thickness (Fig. 6B).

## Discussion

Titanium dioxide NPs have the potential to function as antimicrobial agents with several chemical modifications to optimize their biochemical properties. In this work, we report the synthesis and characterization of TiO_2_ NPs in anatase and rutile forms together with surface modification by GER for antibacterial effect enhancement. The antibacterial and antifungal properties of TiO_2_ against a broad range of pathogens were demonstrated in several previous studies [21] and the use of natural compounds to enhance physical and chemical properties of NPs has also been documented [30, 31, 39]. In our study, GER was chosen as a potential candidate for coupling with TiO_2_ to enhance its antimicrobial properties due to its high solubility and controlled toxicity [40, 41].

The morphology of the TiO_2_ NPs can be tuned by calcination temperature [44]. In line with previous reports [45–47], we successfully synthesized TiO_2_ with two different calcination temperatures, 500 °C confirming the creation of TiO_2_ in the anatase form and 900 °C to generate TiO_2_ in the rutile form. The success of TiO_2_/GER modification was confirmed by the XRD results showing the impact of homogenization on the crystalline structure of the combination [48, 49]. Another verification of the successful modification was done by elemental composition with EDS. Carbon was a definite indicator of response and coverage in the sample. Several earlier studies have detected the presence of carbon, which is attributed to carbon-containing functional groups of natural products [50–52].

Several investigations employing modification of TiO_2_ demonstrated the antibacterial activity of this material [53]. These studies attributed the increase in antimicrobial susceptibility to modifications of TiO_2_ with natural elements and non-metallic compounds [45, 46, 54, 55]. Our MIC results are consistent with previous findings reporting MIC against various G-positive and G-negative bacteria in the similar range [45, 56–59] while other studies reported the MIC against MRSA to be much higher (7.0–13.0 mg/ml) [60, 61]. Our study showed that the UV light on TiO_2_ had no significant changes in the reduction rate. This finding contradicts a previous report that suggested that UV light irradiation of TiO_2_ nanorod arrays exhibited excellent antibacterial properties against both *S. aureus* and *E. coli* biofilm [40].

Although the confocal microscopy results supported the quantitative Alamar blue results, some variation was noticed. In the Alamar blue analysis, both TiO_2_ modifications exhibited greater effectiveness against MRSA biofilms, and neither treatment alone had a statistically significant effect on the biofilms. In contrast, the confocal microscopy indicated that the different TiO_2_ treatments influenced the architecture of the biofilms, as they showed a reduction in biofilm thickness. These findings are supported by previous studies emphasizing the importance of examining biofilms using quantitative and qualitative methods [45]. Therefore, we have demonstrated that TiO_2_ has the potential to act as an anti-biofilm agent on its own without the need for any modification.

The increased antimicrobial activity and reduction in bacterial cell viability due to the exposure of TiO_2_/anatase/GER and TiO_2_/rutile/GER showed that both treatments had a high adsorption capacity on the cell surface. The increased antimicrobial activity of modified TiO_2_ can be explained by several mechanisms. First, geraniol ability to adhere to cell membrane lipids of bacteria can mediate antimicrobial activity by increasing the permeabilization of the membrane [62, 63]. In addition, the presence of TiO_2_, TiO_2_/anatase/GER, and TiO_2_/rutile/GER can lead to loss of cell wall integrity and direct interaction with DNA leading to cell death [64, 65]. Secondly, modifications of NPs are commonly used to prevent aggregation, allowing them to disperse in aqueous environments or other hydrophilic media, thereby enhancing their antimicrobial activity [47]. Lastly, modifications are also one of the most effective methods to regulate and control NPs and bacteria interactions and increase ROS production [31, 32].

Based on these findings, combining TiO_2_ with geraniol, offers an opportunity for the antibacterial use. Low cytotoxic effects, described in Figure S5., highlight the usefulness of these compounds. Nevertheless, further research is needed to find out the full range of potential applications and any additional potential hazards.

## Conclusions

In conclusion, TiO_2_ nanoparticles modified with GER in the form of anatase and rutile were successfully synthesized and tested for their antimicrobial activity. The modified TiO_2_ nanoparticles had the elevated antimicrobial activity and also inhibited MRSA biofilm formation. TiO_2_ alone also proved to be effective against biofilms.

## Methods

### Preparation of TiO_2_ NPs

Titanium isopropoxide (1.48 ml) (Sigma Aldrich in St. Louis, MO, USA) was mixed with 229.0 µl of isopropyl alcohol (Lach-Ner in Neratovice, Czech Republic) and 18.0 µl of MilliQ water (pH 8) (Thermofisher, USA) and stirred for 10 min. Then, the solution was washed three times with MilliQ water and dried overnight in the Memmert UM 400 drying oven (Memmert, Schwabach, Germany) at 60 °C. Finally, TiO_2_ NPs were produced by calcining the dried solution at the temperature of 500 °C for the anatase form and 900 °C for the rutile form for 2 h in the laboratory chamber furnace (LAC in Rajhrad, Czech Republic).

### Modification of TiO_2_ NPs by GER

The TiO_2_ NPs were redispersed in 50 ml of MilliQ water. Then, 10 ml of the dispersion with 161.1 mg of GER (Sigma Aldrich, Saint Louis, USA) was mixed in 200 µl of GER solvent with MilliQ water and stirred overnight. Afterward, the solution was washed three times with MilliQ water.

### Characterization of TiO_2_ NPs

The absorbance wavelength was determined using the UV/Vis spectrophotometer (Perkin-Elmer, USA) with the quartz cuvette with acetonitrile/tetrahydrofuran as a reference. The X-ray powder diffraction patterns of the TiO_2_ NPs were recorded on the Bruker 8D advanced X-ray diffractometer (Bruker, AXS GmbH, Karlsruhe, Germany) with CuKα radiation of wavelength = 1.54056 Å. The morphology of the TiO_2_ NPs was studied by SEM using the JEOL JSM-6330 LA operated at 20.0 kV and 1.0000 nA. The elemental analysis used SEM on a Tescan MIRA 2 equipped with a FEG (Tescan Ltd., Brno, Czech Republic) and EDX detector MAX 50 (Oxford Instruments plc, Abingdon, UK). The images were obtained using the E-T SE detector at a working distance of 15 mm and 15 kV acceleration voltages, with 50 000-fold magnification. Fourier transform infrared spectroscopy (FTIR) analysis was used on a film of each freeze-dried sample using a Jasco FT/IR-4700 (Jasco, MD, USA) spectrophotometer with a wavenumber range of 400 to 4000 cm ^− 1^ with 4 cm ^− 1^ resolutions. The zeta potential and size of particles were measured using the dynamic light scattering technique on the Zeta sizer Nano ZS instrument (Malvern Instrument Ltd, UK). The particle size distribution and ζ-potential were studied using dynamic light scattering (DLS) on the Malvern Zetasizer (NANO-ZS, Malvern Instruments Ltd., Worcestershire, U.K.) at a detector angle of 173° and the refractive index of 1.33 at 25 °C. The size distribution and ζ-potential measurements were performed in MilliQ water at pH 6.8. Measurements were taken for each sample in triplicates.

### Determination of minimum inhibitory concentration (MIC)

Bacterial cultures were obtained from the Czech Collection of Microorganisms (Brno, Czech Republic), including *Staphylococcus aureus* CCM 4223, MRSA CCM 7110, and *Escherichia coli* CCM 3954. All bacterial strains were cultured on Columbia agar with 5% sheep blood (LMS, Czech Republic) at 37 °C for 24 h. The antibacterial activity of TiO_2_/anatase/GER, TiO_2_/rutile/GER, along with control treatments anatase, rutile, and GER, was evaluated against bacterial strains cultured in Muller Hinton broth (company). Bacteria were incubated in the 96 well plate at 37 °C, with 150 rpm shaking; the optical density (OD) at 600 nm was measured after 24 h.

### Determination of fractional inhibitory concentration (FIC)

The effect of the GER and TiO_2_ combination was assessed against bacterial strains using the checkerboard technique [16]. We then calculated the FIC index values using the formula: FICI = FIC (A) + FIC (B). The Σ FICI values were interpreted as follows:

≤ 0.5 = synergistic; >0.5–1.0 = additive; > 1.0–4.0 = indifferent (non-interactive); > 4.0 = antagonistic.

**LIVE/DEAD® Cell Assay**

Live/dead assay was carried out by following the manufacturer instructions of the LIVE/DEAD™ BacLight™ bacterial viability kit for microscopy (Invitrogen, USA). Modified nanomaterials TiO_2_/anatase/GER, TiO_2_/rutile/GER, and non-modified anatase and rutile samples were examined at the defined MIC after 24 h and these bacterial samples were observed by an inverted fluorescent microscope Olympus IX71 (Olympus C&S Ltd., Czech Republic).

### MRSA biofilm assay

The efficacy of TiO_2_ with and without GER agent. MRSA biofilm was evaluated using qualitative and quantitative methods. MRSA was incubated in BHI with 1% glucose for 72 h at 37 °C. Biofilms were treated based on the MIC values, washed twice with PBS, and stained with the alamarBlue™ cell viability reagent (Invitrogen, USA) for 20 min. The 96 well plate was used to carry out the experiment, and biofilm viability was measured using fluorescence measurements (Tecan, Switzerland) (590/570 nm, excitation/emission). Qualitative assessment of the biofilm thicknesses and high-quality visualization images were done using confocal microscopy (LSM 880, USA) with the incubation and washing conditions, with the exception that IBIDI dishes were used and stained using the LIVE/DEAD™ BacLight™ bacterial viability kit for microscopy (Invitrogen, USA).

## Electronic supplementary material

Below is the link to the electronic supplementary material.

Supplementary Material 1

## Data Availability

Data is available on the department share drive and can be uploaded by the first author when requested.

## Abbreviations

NPs: Nanoparticles
GER: Geraniol
SEM: Scanning electron microscopy
EDS: Electron-dispersive spectroscopy
FTIR: Fourier transform infrared spectroscopy
DLS: Dynamic light scattering
MIC: Minimum inhibitory concentration
FIC: Fractional inhibitory concentration
OD: Optical density
MRSA: Methicillin-resistant *Staphylococcus* *aureus*
BHI: Brain heart infusion
PBS: Phosphate-buffered saline
