# Supplementary material for: Synthesis and characterization of TiO2 nanoparticles combined with geraniol and their synergistic antibacterial activity
Source: BMC Microbiol. 2023 Aug 2;23:207. doi: 10.1186/s12866-023-02955-1 (PMC10394861; doi:10.1186/s12866-023-02955-1)
Supplement: Supplementary file 1 — Supplementary Material 1 [file 12866_2023_2955_MOESM1_ESM.docx]

**Supplementary Materials and Methods**

An *in vitro* MTT assay was used to assess the cytotoxic effects of geraniol and titanium dioxide nanoparticles (in anatase and rutile forms) and their combination form with geraniol. A549 lung cells were cultured on 96-well plates and then exposed to a variety of geraniol, anatase, and rutile concentrations (0.1, 0.25, and 0.5 mg/ml) as well as mixes of rutile and geraniol and anatase. These substances were given to the cells to interact with for 24 hours. After this exposure, phosphate-buffered saline (PBS) was used to wash the cells clean of these substances. The cells were then exposed to an MTT solution (0.5 mg/ml) in the full medium and incubated at 37°C for 3 hours. To remove the MTT solution, another round of washing with PBS was then performed. The cells were then exposed to DMSO in order to dissolve the generated formazan crystals. Finally, a microplate reader was used to measure the absorbance at 560 nm and 670 nm in order to quantify the resulting color change.

Figure S1. Baseline-corrected FTIR spectra of geraniol.


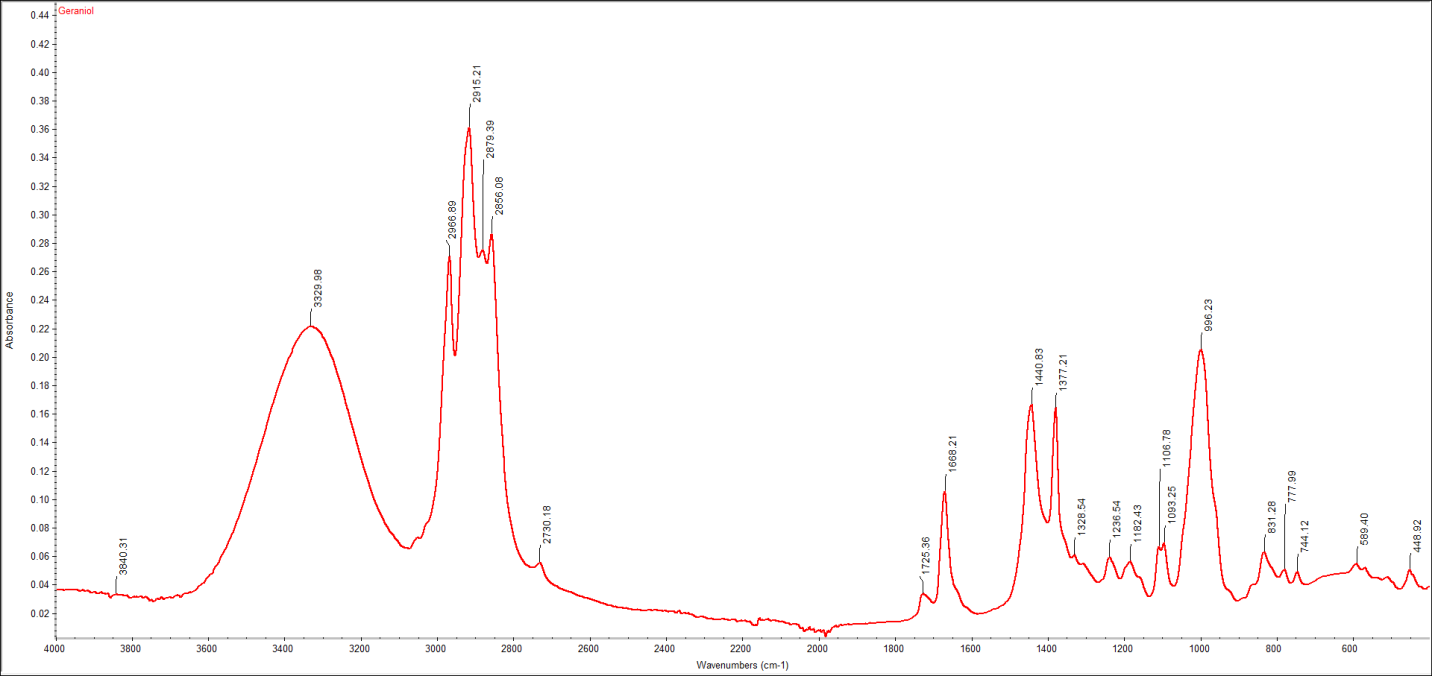


Figure S2. Zeta sizer of TiO_2_ and its combination with geraniol. The larger particles of anatase and rutile may have less antimicrobial activity due to less surface area to interact with the microbes. Conversely, the smaller particles of anatase/geraniol and rutile/geraniol may exhibit more antimicrobial activity due to the increased surface area. The combination of materials with geraniol (anatase/geraniol and rutile/geraniol) might have altered the particle size distribution, influencing their antimicrobial activity (1-3).





Figure S3. Zeta potential of TiO_2_ nanoparticles synthesized and combinations with geraniol. The zeta potential values in our characterization experiments show various dispersion stabilities. Due to their small negative charges, geraniol and rutile TiO_2_ suggest stable dispersions. Although positively charged TiO_2_ anatase may interact differently, stability should still be achieved. The charge of the rutile/geraniol composite was the most negative, indicating the most stability, but the charge of the anatase/geraniol composite suggests slightly lower stability. These discoveries help understanding the behaviour of these compounds (4-6).


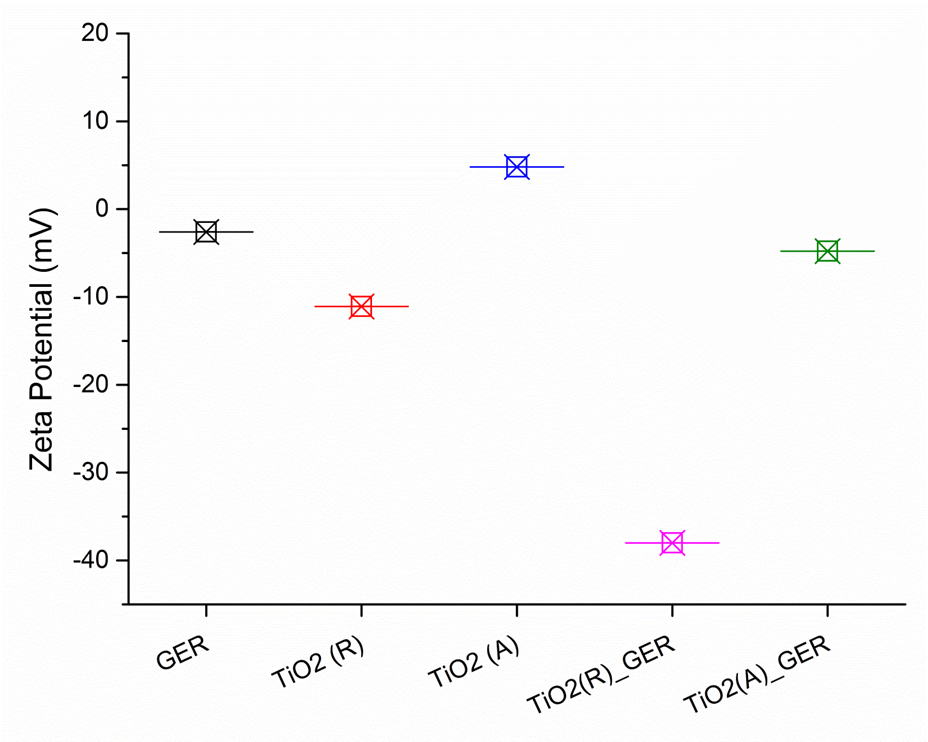


**MTT assay, and isobologram**

This file presents the data from the MTT assay, the FTIR characterization of geraniol, and the isobologram figures demonstrating synergistic effects of TiO_2_ and geraniol combinations. The MTT assay performed on healthy lung cells evaluated the cytotoxicity of different treatments. The treatments comprised TiO_2_ nanoparticles in anatase and rutile forms, geraniol, and their combination at various concentrations (0.1, 0.25, 0.5 mg/ml). Overall, the treatments showed a dose-dependent impact on cell viability. In the case of anatase, a moderate reduction in cell viability was observed at higher concentrations, with the average cell viability being 21% and 38% at concentrations of 0.5 µg/mL and 0.1 mg/mL, respectively. However, rutile, at the same concentrations, showed cell viability of 43% and 60%, indicating a comparatively lesser cytotoxic effect than anatase (3, 7). Geraniol showed a similar pattern, with cell viability ranging from 34% to 100%, depending on the concentration(8). Interestingly, the combination of anatase and geraniol showed improved cell viability, particularly at lower concentrations, with averages ranging from 43% to 72%. Similarly, the combination of rutile and geraniol resulted in average cell viability ranging from 48% to 70%. These results suggest that while individual treatments may present some cytotoxic effects at higher concentrations, their combinations, particularly with geraniol, appear to mitigate this effect, offering potentially safer antimicrobial solutions.

**Figure S4. Isobologram for determination of synergy
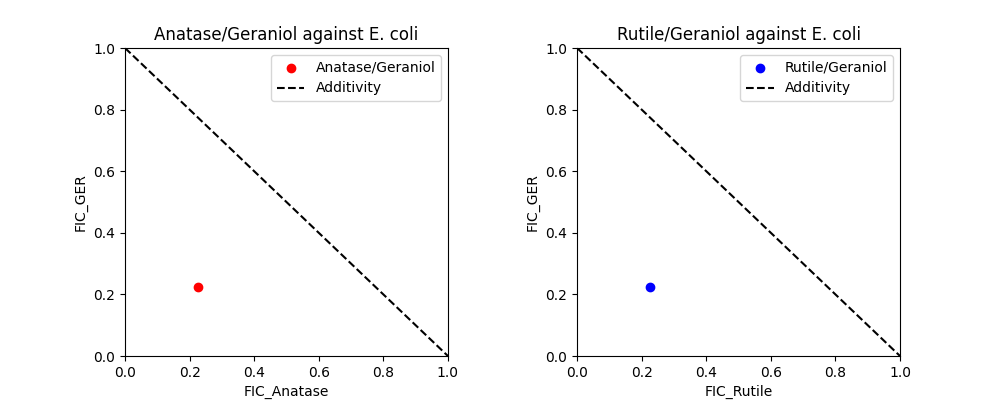
**
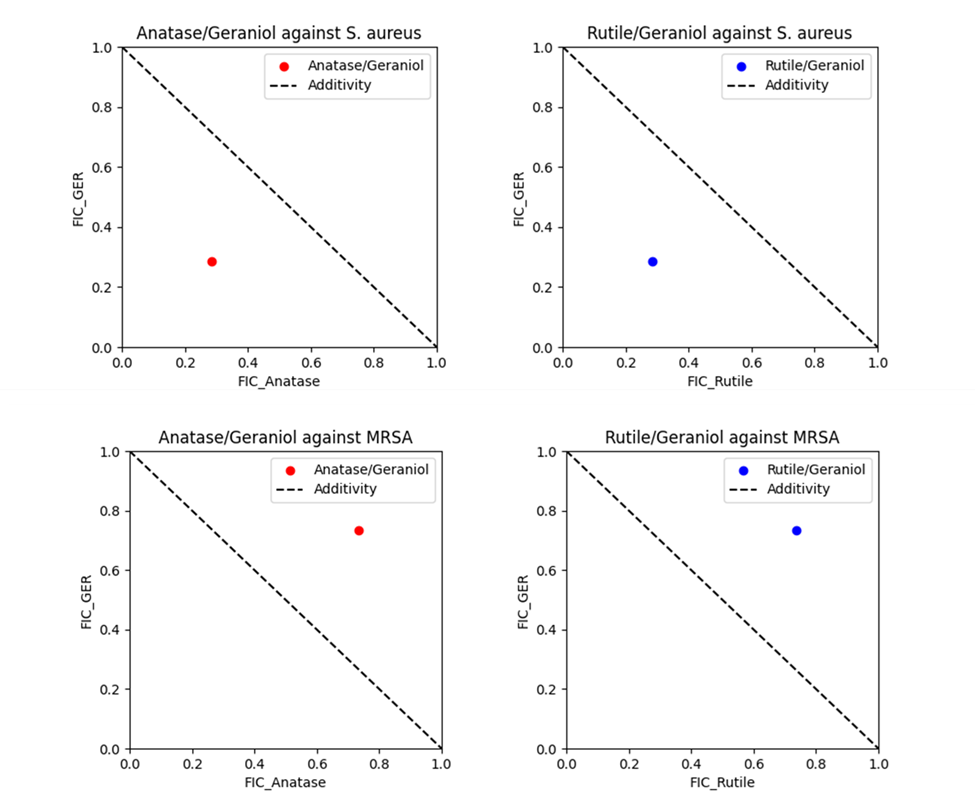


Figure S5. Cell viability of A549 lung cells after different treatments.


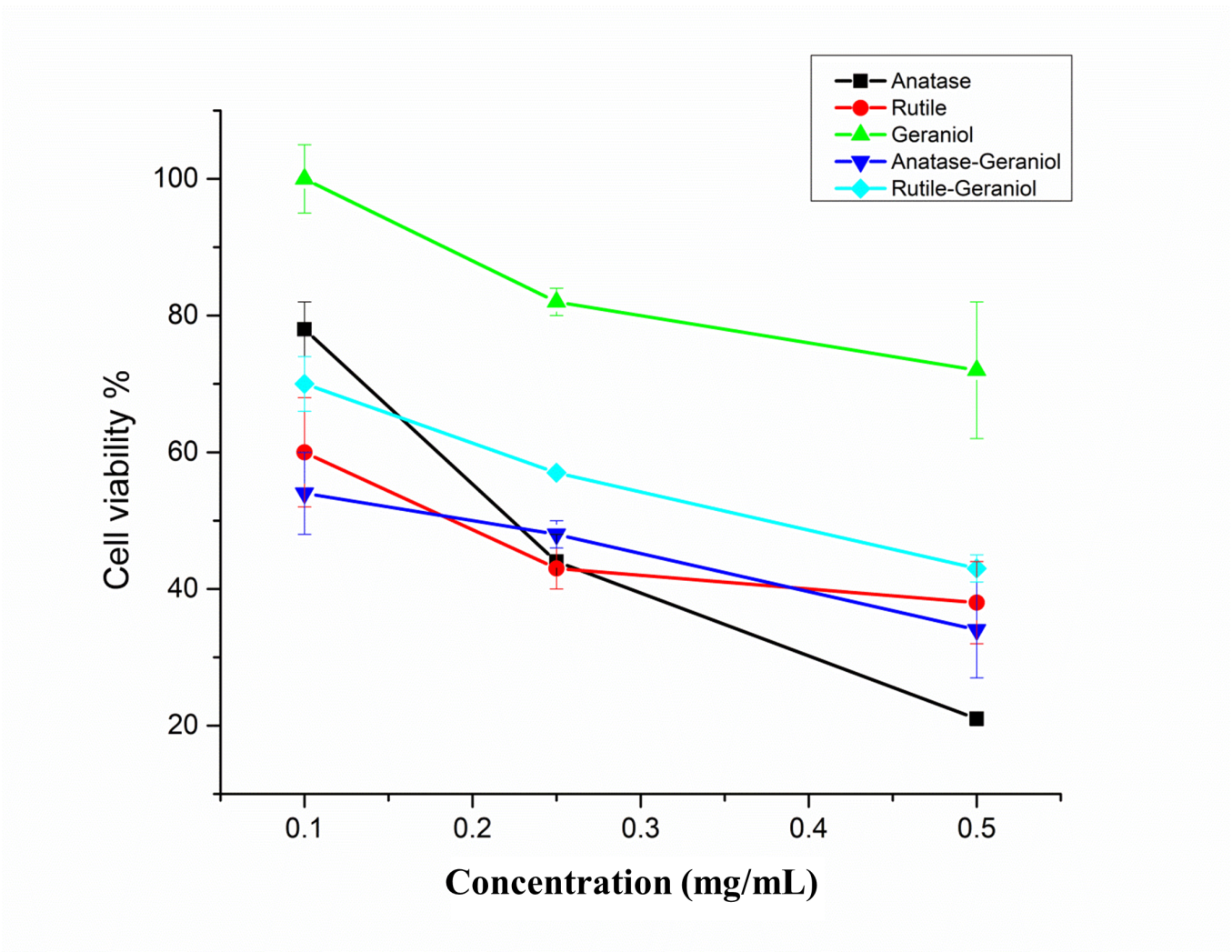


**Table S1. Optical density (OD600nm) measurements reflecting the efficacy of TiO_2_ and geraniol combinations against MRSA, *Staphylococcus aureus*, and *Escherichia coli*.**


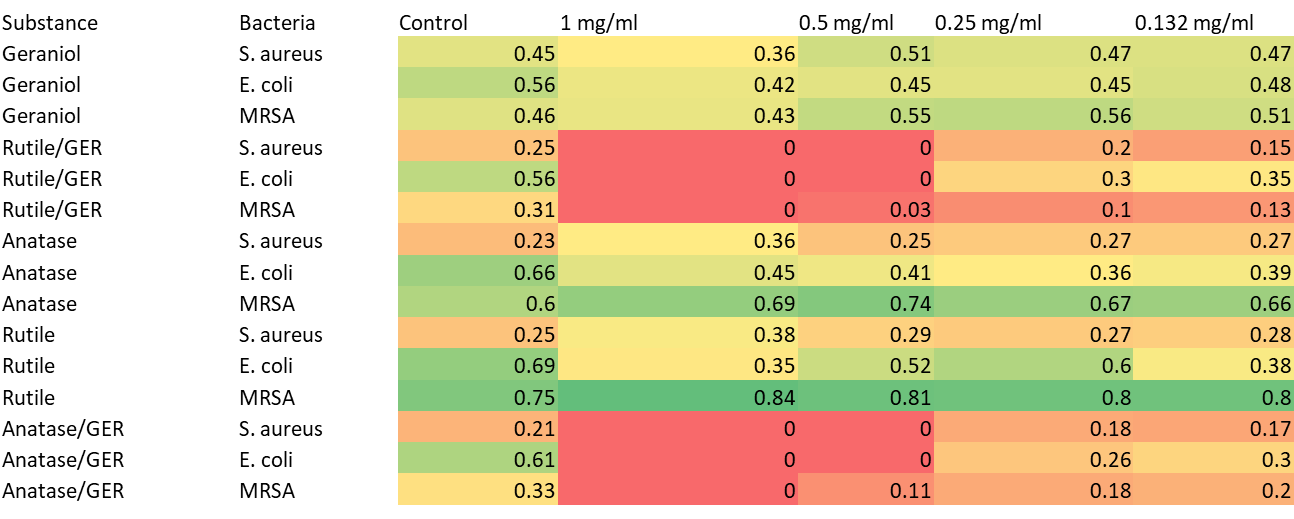


Table S2. Reduction rates of *Escherichia coli*, MRSA, and *Staphylococcus aureus* for various concentrations of TiO_2_ and geraniol combinations.


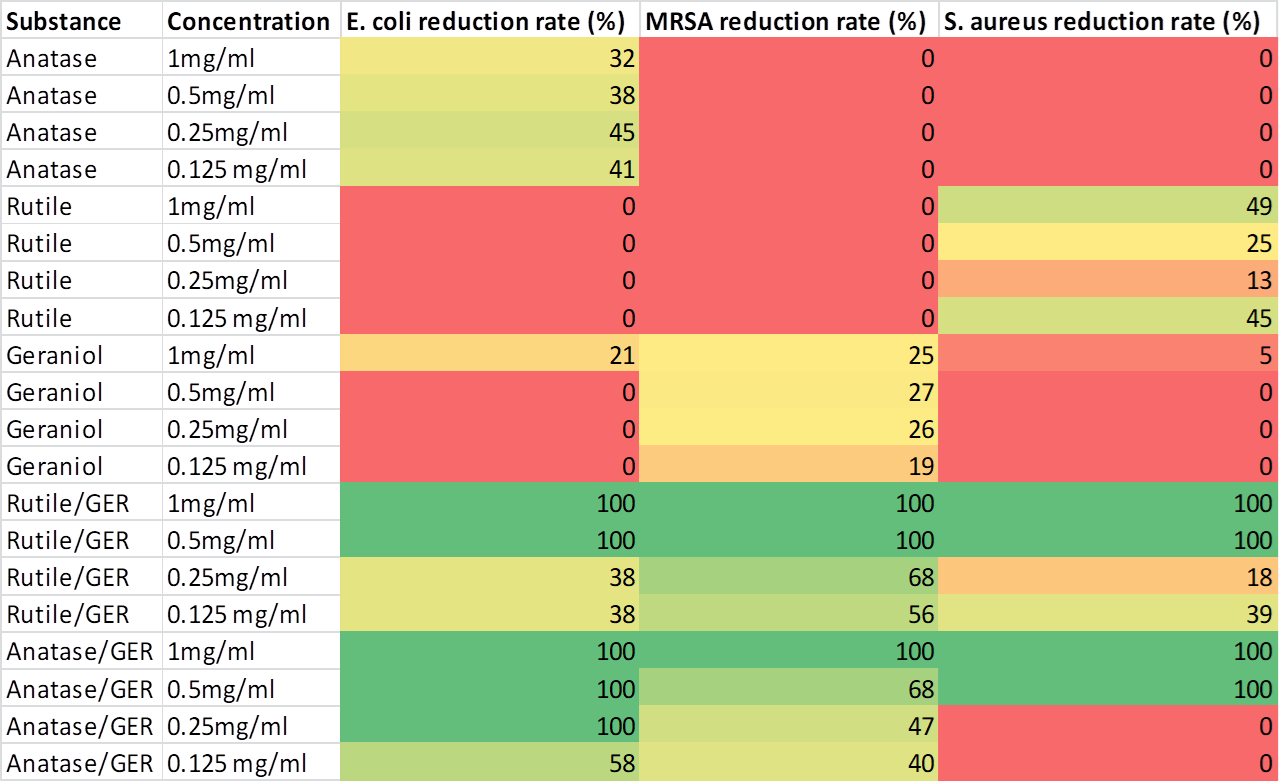


**Supplementary references**

1. Krumdieck SP, Boichot R, Gorthy R, Land JG, Lay S, Gardecka AJ, et al. Nanostructured TiO2 anatase-rutile-carbon solid coating with visible light antimicrobial activity. Sci Rep. 2019;9(1):1883.

2. Dokan FK. Investigation of the Effect of Different Synthesis Methods on the Photocatalytic Activity of TiO2: Comparison of Rutile and Anatase TiO2. Cumhuriyet Science Journal. 2022;43(3):409-15.

3. Guichard Y, Schmit J, Darne C, Gaté L, Goutet M, Rousset D, et al. Cytotoxicity and genotoxicity of nanosized and microsized titanium dioxide and iron oxide particles in Syrian hamster embryo cells. Annals of Occupational Hygiene. 2012;56(5):631-44.

4. Ansari MA, Kalam A, Al-Sehemi AG, Alomary MN, AlYahya S, Aziz MK, et al. Counteraction of biofilm formation and antimicrobial potential of Terminalia catappa functionalized silver nanoparticles against Candida albicans and multidrug-resistant Gram-negative and Gram-positive bacteria. Antibiotics. 2021;10(6):725.

5. Muenraya P, Sawatdee S, Srichana T, Atipairin A. Silver nanoparticles conjugated with colistin enhanced the antimicrobial activity against gram-negative bacteria. Molecules. 2022;27(18):5780.

6. Goswami AM, Sarkar TS, Ghosh S. An Ecofriendly synthesis of silver nano-bioconjugates by Penicillium citrinum (MTCC9999) and its antimicrobial effect. Amb Express. 2013;3(1):1-9.

7. Biola-Clier M, Béal D, Caillat S, Libert S, Armand L, Herlin-Boime N, et al. Comparison of the DNA damage response in BEAS-2B and A549 cells exposed to titanium dioxide nanoparticles. Mutagenesis. 2017;32(1):161-72.

8. Queiroz T, Santos G, Ventura S, Hiruma-Lima C, Gaivã I, Maistro E. Cytotoxic and genotoxic potential of geraniol in peripheral blood mononuclear cells and human hepatoma cell line (HepG2). Genetics and Molecular Research. 2017;16(3).
